# N6-Methyladenosine Modification of LncRNA DUXAP9 Promotes Renal Cancer Cells Proliferation and Motility by Activating the PI3K/AKT Signaling Pathway

**DOI:** 10.3389/fonc.2021.641833

**Published:** 2021-06-08

**Authors:** Lei Tan, Yiming Tang, Hongbo Li, Pengju Li, Yunlin Ye, Junjie Cen, Chengpeng Gui, Junhang Luo, Jiazheng Cao, Jinhuan Wei

**Affiliations:** ^1^ Department of Urology, The First Affiliated Hospital of Sun Yat-Sen University, Guangzhou, China; ^2^ Department of Musculoskeletal Oncology, The First Affiliated Hospital of Sun Yat-Sen University, Guangzhou, China; ^3^ Department of Urology, Sun Yat-Sen University Cancer Center, Guangzhou, China; ^4^ Department of Urology, Jiangmen Central Hospital, Affiliated Jiangmen Hospital of Sun Yat-sen University, Jiangmen, China

**Keywords:** N6-methyladenosine, lncRNA, ccRCC, EMT, Akt

## Abstract

Most localized human renal clear cell carcinoma (ccRCC)-related deaths result from cancer recurrence and metastasis. However, the precise molecular mechanisms largely remain unknown. In recent years, an increasing number of long noncoding RNAs (lncRNAs) have been shown to be vital regulators of tumorigenesis. In this study, we characterized a lncRNA DUXAP9 and the upregulation of DUXAP9 was analyzed by quantitative real-time PCR in 112 pairs of localized ccRCC tumor tissues compared with adjacent normal tissues. Kaplan–Meier curves showed that patients of localized ccRCC with high DUXAP9 expression had poorer overall survival (P<0.01) and progression-free survival (P<0.05) than cases with low DUXAP9 expression. Multivariate Cox regression analysis also showed that high DUXAP9 expression was an independent risk factor for poor prognosis in localized ccRCC (p<0.05). DUXAP9 knockdown in renal cancer cells inhibited renal cancer cells proliferation and motility capacities *in vitro* and reversed epithelial–mesenchymal transition (EMT), whereas overexpression of DUXAP9 promoted renal cancer cells proliferation and motility capacities *in vitro* and induced EMT. Pull-down, RNA immunoprecipitation and RNA stability assays (involving actinomycin D) showed that DUXAP9 was methylated at N6-adenosine and binds to IGF2BP2, which increases its stability. DUXAP9 activate PI3K/AKT pathway and Snail expression in renal cancer cells. DUXAP9 may be useful as a prognostic marker and/or therapeutic target in localized ccRCC.

## Introduction

Renal cell carcinoma (RCC) is the seventh most common cancer in the world (1, 2). As abdominal imaging is gradually becoming more common, so is coincidental detection of renal masses. In high-income countries, the incidence of RCC is increasing (3). There has been a substantial improvement in treatment over the decades. However, a notable proportion of patients have locally advanced disease or distant metastases at diagnosis. Clear cell renal cell carcinoma (ccRCC), the most common RCC subtype (accounting for 85–90% of cases) kills more than 140 000 individuals per year. The conventional therapy for ccRCC is surgical resection or ablation. Nevertheless, around 30% of patients with localized ccRCC develop recurrence or metastasis after this treatment (1, 3, 4). Compared to other tumors, ccRCC is highly insensitive to conventional chemotherapy and radiation (4). Therefore, it is particularly important and urgent to target certain oncogenes and the tumorigenic pathways that drive ccRCC cell proliferation and metastasis, which represents the optimal therapeutic strategy to prevent recurrence and metastasis. Although the recent introduction of molecularly targeted agents has modestly improved the prognosis of ccRCC patients, uncertainties and controversies remain. Further research is expected to bring about further progress.

lncRNAs form a large class of ncRNAs that are >200 nucleotides in length. Not all functional ncRNAs lose their ability to encode peptides (5, 6) Increasing studies have demonstrated the existence of bifunctional transcripts that are not only protein templates but also act as lncRNAs. They can act as key regulators of various cellular processes under physiological conditions as well as in multiple diseases, including the development and progression of cancers, in humans. A great deal of data has shown strong associations between certain lncRNAs and poor prognosis in various types of human cancer (7). lncRNAs are precisely regulated, and they can control gene expression to regulate various aspects of physiological and/or pathological processes (5, 8). They are involved in many important cancer phenotypes, including proliferation, angiogenesis, apoptosis, and immortality (8, 9). For instance, the HOTAIR and LNMAT1 lncRNAs recruit tumor-derived functional proteins, which contribute to immune evasion, proliferation, invasion, migration, and angiogenesis in cancer (10, 11). Nevertheless, functions of lncRNAs and underlying molecular mechanisms in renal cancer remain largely unclear and require further elucidation.

Here, we identified a lncRNA DUXAP9 (ENSG00000225210) which was dramatically upregulated in localized ccRCC. Additionally, DUXAP9 expression was significantly associated with the overall survival (OS) and progression-free survival (PFS) of localized ccRCC patients. DUXAP9 was methylated at N6-adenosine and binds to IGF2BP2, which increases its stability. DUXAP9 activate PI3K/AKT pathway and Snail expression in renal cancer cells. This study provides a more detailed understanding of DUXAP9 as a new potential target for the diagnosis and treatment of renal cancer.

## Materials and Methods

### Patients and Clinical Characteristics

First, 112 pairs of primary localized ccRCC and matched adjacent non-tumor tissues (obtained from December 2007 to October 2018) in the Sun Yat-Sen University Cancer Center (SYSUCC) Biobank were assessed by quantitative real time polymerase chain reaction (qPCR). Pathological diagnosis, clinical, and follow-up data were available for all 112 cases. The median follow-up time was 78.4 months (range: 3.0–143.6 months). The overall 3‐year survival rate was 0.89. The overall 5‐year survival rate was 0.87. The overall 7‐year survival rate was 0.85. The ccRCC tumor, node, metastasis (TNM) status was assessed according to the Journal of European Urology and AJCC/UICC guidelines (2017). The histopathological grade was assessed according to Fuhrman classification. The prognostic value of DUXAP9 expression was analyzed by Kaplan–Meier analysis and univariate and multivariate Cox regression. The analysis of the tissue samples was conducted according to the provisions of the Declaration of Helsinki and was approved by the Ethics Committee of SYSUCC.

For validation, lncRNA expression data and detailed clinical data of 445 patients with localized ccRCC were obtained from The Cancer Genome Atlas (TCGA) database (https://cancergenome.nih.gov/). These data were analyzed in R, which is a free software for statistical computing and graphics. Global gene expression profiles of localized ccRCC tissues, with valid DUXAP9 expression data, in the TCGA database were used in a gene set enrichment analysis (GSEA; http://www.broadinstitute.org/gsea/) to assess the associations between DUXAP9 and cell signaling pathways (in particular, the Akt signaling pathway).

### Cell Lines and Cell Culture

Five renal cancer cell lines (769-P, 786-O, Caki-1, Umrc6, and ACHN) from the Chinese Academy of Sciences were cultured in Dulbecco’s Modified Eagle’s Medium (DMEM; Gibco) with 10% fetal bovine serum (FBS) containing 1% penicillin–streptomycin. Normal human kidney (HK)-2 cells were cultured in DMEM/F12 (Gibco) with the same supplement (12, 13). The cells were cultured at 37°C in 5% CO_2_ in an incubator.

### Quantitative Real-Time PCR

Total RNA was extracted using TRIzol reagent (Invitrogen) and quantified as previously described (12, 13). PrimeScript Master Mix (RR036A; TaKaRa) was used for reverse transcription following the manufacturer’s instructions. qRT-PCR was performed on a standard LightCycler 480 Real-Time PCR System (Roche, Indianapolis, IN, USA) using an SYBR FAST Universal qPCR Kit (Roche). Firstly, the Ct values of internal reference genes were used to standardize the Ct values of target genes for all samples. Secondly, Δ Δ CT value was obtained by subtracting the standardized Ct values of target genes from normal tissues from the standardized Ct values of target genes from tumor tissues. Finally, relative expression levels (fold changes) were calculated using the 2^-ΔΔCt^ method. Primers are listed in **Supplementary Data 1**.

### Western Blotting

Total proteins were extracted using radioimmunoprecipitation assay (RIPA) lysis buffer (Beyotime, China) containing a proteinase inhibitor cocktail (Roche, USA). The protein sample was then quantified using a bicinchoninic acid (BCA) protein assay kit (Thermo Fisher Scientific, USA). The proteins were separated using sodium dodecyl sulfate polyacrylamide gel electrophoresis (SDS-PAGE) and transferred to a nitrocellulose membrane. After blocking and incubating with primary antibody followed by secondary antibody, the membranes were observed using a ChemiDoc™ Touch Imager (BIO-RAD, USA). Primary antibodies against the following proteins were used: cleaved caspase-8, cleaved caspase-3, cleaved PARP, Akt, PI3K, phospho (p)-Akt, mammalian target of rapamycin (mTOR), p-mTOR, GSK3β, p-GSK3β and EMT proteins (including N-cadherin, E-cadherin, vimentin, and Snail) assessed by an EMT Antibody Sampler Kit (Cell Signaling Technology, Beverly, MA, USA), IGF2BP2, METTL3, Bcl-2, Bax, and β-actin (Proteintech Group, Inc., Rosemont, USA). The secondary antibodies were horseradish peroxidase (HRP)-conjugated anti-mouse and anti-rabbit antibodies (Proteintech Group, Inc.).

### 5’ and 3’ Rapid Amplification of cDNA Ends

5’ and 3’ RACE using primers specific to the DUXAP9 transcript was conducted in Caki-1 cells using a SMARTer RACE 5/3 kit (Takara Biomedical Technology, Japan) following the manufacturer’s instructions. The sequences for the DUXAP9-specific nested PCR primers are shown in **Supplementary Data 1**.

### RNA Fluorescence *In Situ* Hybridization (FISH) and Subcellular Fractionation Assays

CY3-labeled FISH probes for DUXAP9, U6, and 18S were synthesized by Ribo Bio (Guangzhou, China). A FISH kit (Ribo Bio) was utilized according to the manufacturer’s protocol. Briefly, 2×10^4^ Caki-1cells were seeded in plates for confocal microscopy, fixed with 4% paraformaldehyde, treated with 0.5% Triton X-100 in ice-cold phosphate-buffered saline (PBS), prehybridized, and then hybridized overnight with 5 μM CY3-labeled FISH probe. Fluorescence confocal microscopy (Nikon, Japan) was used to directly detect the cellular location of DUXAP9. Additionally, nuclear and cytoplasmic fractions were separated and the level of DUXAP9 in each was assessed using a PARIS™ Protein and RNA Isolation System (Invitrogen, USA) following the manufacturer’s instructions.

### Flow Cytometry Analysis of Apoptosis

To analyze the apoptosis rate, preconditioned cells were processed using an Annexin V Alexa Fluor 647/propidium iodide (PI) kit (4Abio, Beijing, China) according to the manufacturer’s instructions. Briefly, the cells were digested with ethylenediaminetetraacetic acid (EDTA)-free trypsin, centrifuged, washed in precooled PBS, and resuspended in binding buffer. The cells were then stained with Annexin V Alexa Fluor 647 and PI and analyzed using a flow cytometer (CytoFLEX, Beckman Coulter, Brea, CA, USA).

### Cell Counting Kit-8 and Colony Formation Assays

Cell proliferation was assessed using a CCK-8 assay (Beyotime Technology, China). Briefly, 1,000 cells were seeded in 96-well plates and the absorbance was assessed every 24 h following the manufacturer’s instructions. Additionally, colony formation assays were performed with 500 cells seeded in 6-well plates. After 7 days, the colonies were stained using crystal violet. Each assay was performed in triplicate.

### Wound Healing Assays

To assess cell migration, cells were cultured to produce a monolayer, which was scratched using a sterile 200-mL pipette tip. Wound healing was assessed after image acquisition by measuring the gap area in each image.

### Migration and Invasion Assays

In vitro migration and invasion assays were performed in a 24-well Boyden Chamber (Corning Inc., Corning, NY, USA) and a BioCoat Matrigel Invasion Chamber (BD Biosciences, Bedford, USA) according to the manufacturers’ instructions. The membranes were coated with Matrigel matrix in the invasion but not the migration assays. In both assays, 5×10^4^ cells were seeded in the upper chamber with serum-free medium. Medium with 20% FBS was added to the lower chamber. After 24 h, the membranes, with invaded cells, were collected. The migrated and invaded cells were fixed with paraformaldehyde, stained using 5% crystal violet, and counted under a microscope. Each assay was performed in triplicate.

### Knockdown and Overexpression

Small interference RNA (siRNA) of IGF2BP2 and METTL3 and corresponding negative control were chemically synthesized by RiboBio (RiboBio, Guangzhou, China) for further study (14). Lentiviral packaging plasmids encoding a short hairpin RNA (shRNA) targeting DUXAP9 (shRNA1 or shRNA2) and the negative control plasmids were obtained from GeneCopoeia (Rockville, USA). The shRNA target sequence (10) is shown in **Supplementary Data 1**. To produce the lentiviruses, 293T cells were used as previously described (12). A recombinant plasmid overexpressing DUXAP9 was synthesized and constructed by our group using the DUXAP9 transcripts from the 5’ and 3’ RACE experiments. renal cancer cells were transfected with the recombinant plasmids using Lipofectamine 2000 (Thermo Fisher Scientific) following the manufacturer’s protocol. Puromycin (2 µg/mL) was used to select the cells with modified DUXAP9 expression and qPCR was used to quantify the efficiency of plasmids.

### RNA Immunoprecipitation–qPCR Assays

RIP-qPCR assays were performed using a Magna RIP RNA-Binding Protein Immunoprecipitation Kit (17-700; Millipore), according to the manufacturer’s instructions. Briefly, Caki-1 cell lysates were incubated overnight at 4°C with Protein-A/G beads (Roche) and 5 μg of IGF2BP2-specific antibody, m6A-specific antibody, or control IgG antibody. The resulting immune complexes were washed six times with washing buffer and incubated with proteinase K digestion buffer. Lastly, total RNA was extracted and analyzed by qPCR (normalized to the input).

### RNA Pull-Down Assays

The proteins that bind to DUXAP9 were investigated using RNA pull-down assays using a Pierce Magnetic RNA-Protein Pull-Down Kit (Thermo Fisher Scientific) according to the manufacturer’s instructions. Biotinylated wildtype DUXAP9, an antisense control sequence, and m6A (GAACT) motif-mutated DUXAP9 were synthesized using a T7 Transcription Kit (Thermo Fisher Scientific). The proteins in the RNA–protein complex in the pull-down assays were identified by western blotting and mass spectrometry.

### Mutation of m6A Motif in DUXAP9

The potential m6A sites in DUXAP9 were predicted using SRAMP (http://www.cuilab.cn/sramp/). Thereafter, the m6A (GAACT) motif in DUXAP9 was mutated. Cloning into pcDNA3.1 was then conducted for the RNA pull-down assays detailed in *RNA Pull-Down Assays*.

### RNA Stability Assays

To explore whether IGF2BP2 affect the stability of DUXAP9 in renal cancer cells, we knocked down IGF2BP2 and METTL3 individually in Caki-1 and Umrc6 cells and assessed DUXAP9 expression. After inhibiting further transcription by treating the Caki-1 and Umrc6 cells with actinomycin D (final concentration, 5 μg/mL), total RNA was extracted from the cells at 0, 3, and 6 h using TRIzol reagent (Invitrogen, Carlsbad, USA). The half-life of DUXAP9 was then analyzed as previously described (15, 16).

### Statistical Analysis

The data were analyzed using SPSS 20.0 (IBM, Armonk, NY, USA) and GraphPad Prism 6.0 (GraphPad Software, La Jolla, CA, USA). Student’s t-test was used to compare differences in continuous variables between two groups. The error bars indicate the standard deviation (SD) from three independent experiments. Survival analysis was carried out using Kaplan–Meier analysis and the log-rank test. Univariate and multivariate Cox regression analyses was used to assess DUXAP9 expression associations with OS and PFS. P<0.05 was considered to represent statistical significance.

## Results

### DUXAP9 Was Frequently Upregulated in Localized ccRCC and Positively Associated With Poor Outcomes

**Figure 1:**
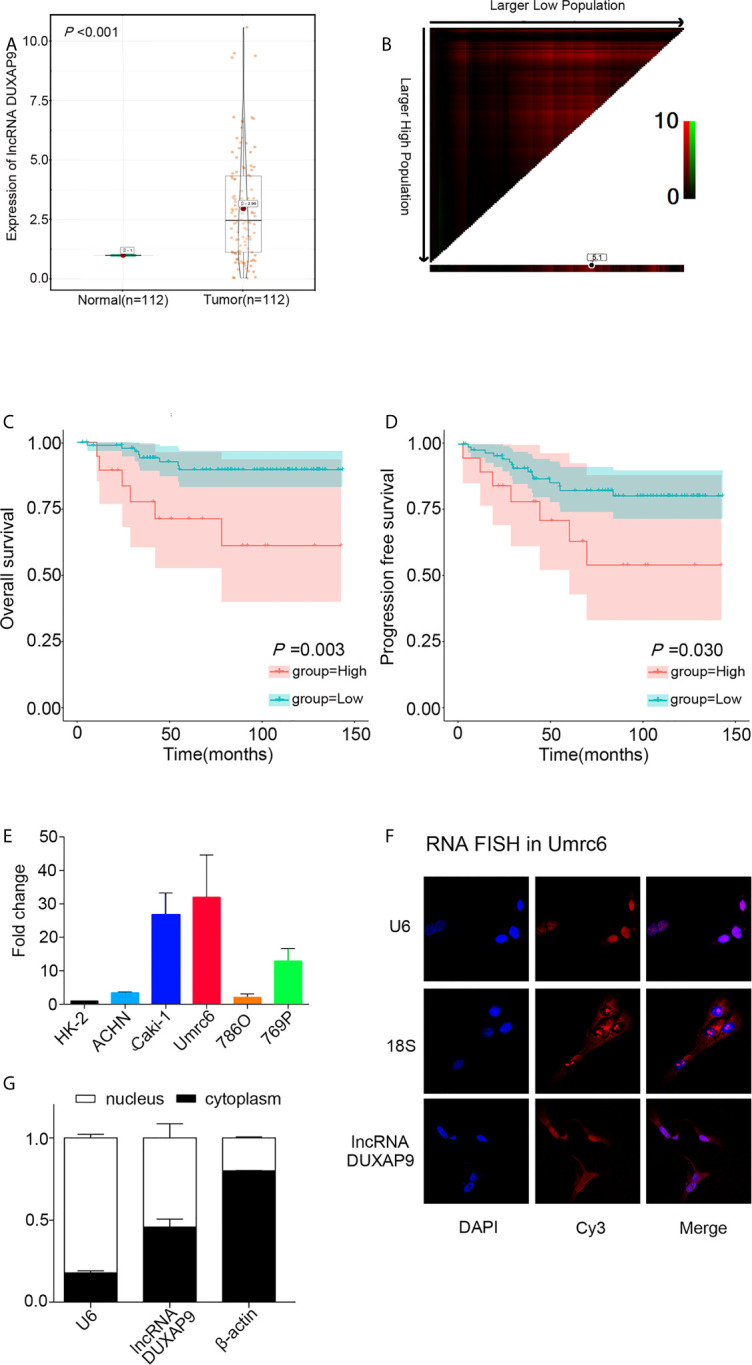
DUXAP9 was upregulated in renal cancer and predicted poor prognosis in localized ccRCC patients. **(A)** Relative DUXAP9 expression levels in 112 pairs of localized ccRCC and matched adjacent non-tumor tissues from the SYSUCC Biobank, as detected by qRT-PCR. **(B)** Optimal cutoff to divide ccRCC patients into high and low DUXAP9 expression groups, as determined by X-tile bioinformatics software based on the integral optic density. **(C, D)** Kaplan–Meier curves showing that high DUXAP9 expression (n=19) in localized ccRCC tissues was significantly associated with poor OS and PFS rates, compared with low expression (n=93). **(E)** Relative DUXAP9 expression levels in renal cancer cell lines, as detected by qRT-PCR. **(F)** FISH analysis of the subcellular distribution of DUXAP9 in Umrc6. **(G)** Subcellular fractionation and qRT-PCR analyses of DUXAP9 expression in the nucleus and cytoplasm. Data represent mean ± SD from three independent experiments. ccRCC, clear cell renal cell carcinoma; qRT-PCR, quantitative real-time PCR; OS, overall survival; PFS, progression-free survival; FISH, fluorescence *in situ* hybridization.

We first examined DUXAP9 expression in 112 pairs of primary localized ccRCC and adjacent non-tumor tissues from SYSUCC Biobank. The results showed a consistent tendency for DUXAP9 expression to be increased in ccRCC tissues compared to adjacent non-tumor tissues (P<0.001) (**Figure 1A**). We obtained a cutoff to divide these patients into high and low expression groups using X-tile software (based on the integral optic density) (**Figure 1B**). Furthermore, the Kaplan–Meier analysis demonstrated that upregulated DUXAP9 was significantly associated with poor OS and PFS (P=0.003 and P=0.030, respectively) (**Figures 1C, D** **)**. Multivariate Cox regression of tissues from SYSUCC Biobank confirmed that upregulated DUXAP9 was an independent risk factor for poor OS (hazard ratio [HR]: 4.560, P=0.010) in localized ccRCC patients (**Table 1**). We then investigated DUXAP9 expression in the RCC cell lines using qRT-PCR (**Figure 1E**). Endogenous DUXAP9 was overexpressed in the five RCC cell lines compared to HK-2 cells which are proximal tubule cells obtained from a normal kidney (13). Umrc6 and Caki-1 cell lines had obviously higher DUXAP9 expression in contrast to the others. FISH and subcellular fractionation assays were used to confirm that DUXAP9 was not only localized to the nucleus but also the cytoplasm in the Umrc6 cells (**Figures 1F, G** **)**.

**Table 1:** Univariate and multivariate analyses of factors predicting overall survival among localized ccRCC cases from SYSUCC Biobank.

Factor	Univariate analysis		Multivariate analysis	
	HR (95% CI)	P value	HR (95% CI)	P value
Age (60 years or older vs younger than 60 years)	2.817 (0.988–8.033)	0.053	1.345 (0.381–4.745)	0.645
Fuhrman grade (G4 vs G3 vs G2 vs G1)	3.016 (1.538–5.912)	**0.001**	3.067 (1.405–6.695)	**0.005**
Stage (III vs II vs I)	1.809 (0.984–3.324)	0.056	1.173 (0.616–2.234)	0.627
DUXAP9 expression (High vs Low)	4.414 (1.528–12.751)	**0.006**	4.560 (1.440–14.438)	**0. 010**

ccRCC, clear cell renal cell carcinoma; SYSUCC, Sun Yat-Sen University Cancer Center; HR, hazard ratio; CI, confidence interval. The bold numbers indicate the P-values with statistical significance.

**Figure 2:**
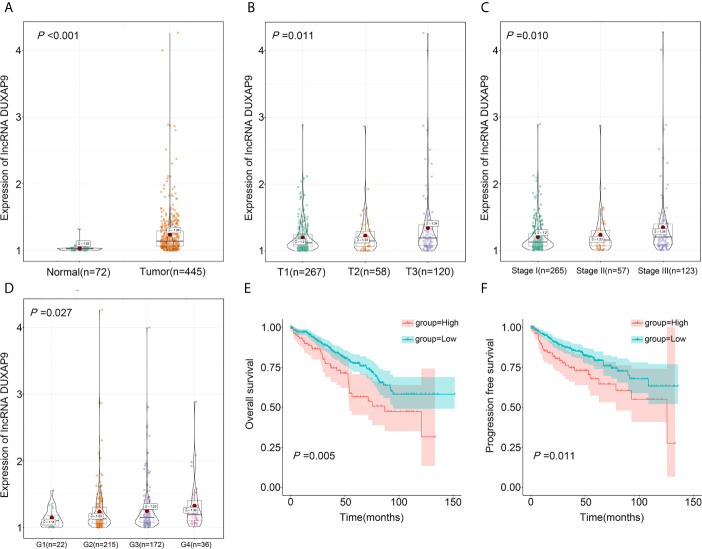
DUXAP9 was identified as an oncogenic factor and predicted poor prognosis in localized ccRCC patients in the TCGA database. **(A)** DUXAP9 expression in 445 localized ccRCC tissues and 72 normal tissues in the TCGA database. **(B–D)** DUXAP9 expression increased with increasing T classification, stage, and Furman grade. **(E, F)** Kaplan–Meier analyses confirmed that high DUXAP9 expression (n=103) in localized ccRCC tissues was significantly associated with poor OS and PFS, compared with low expression (n=342). ccRCC, clear cell renal cell carcinoma; TCGA, The Cancer Genome Atlas; OS, overall survival; PFS, progression-free survival.

To verify the above results, we analyzed TCGA data on DUXAP9 expression in 445 localized ccRCC tissues and 72 normal renal tissues. DUXAP9 was significantly upregulated in localized ccRCC compared to normal renal tissues (P<0.001) (**Figure 2A**). In addition, as shown in the violin plots in **Figures 2B–D**, DUXAP9 expression in localized ccRCC increased with increasing T classification (P=0.011), stage (P=0.010), and grade (P=0.027). Moreover, DUXAP9 upregulation was associated with poor OS and PFS (P=0.005 and P=0.011, respectively) (**Figures 2E, F** **)**. Multivariate Cox regression of the TCGA data confirmed that upregulated-DUXAP9 was an independent risk factor for poor OS in localized ccRCC patients (HR: 1.767, P=0.006) (**Table 2**).

**Table 2:** Univariate and multivariate analyses of factors predicting overall survival among localized ccRCC cases from TCGA.

Factor	Univariate analysis		Multivariate analysis	
	HR (95% CI)	P value	HR (95% CI)	P value
Age (60 years or older vs younger than 60 years)	2.373 (1.548–3.640)	**<0.001**	2.101 (1.367–3.229)	**<0.001**
Fuhrman grade (G4 vs G3 vs G2 vs G1)	1.851 (1.415–2.420)	**<0.001**	1.540 (1.168–2.031)	**0.002**
Stage (III vs II vs I)	1.633 (1.325–2.011)	**<0.001**	1.374 (1.102–1.713)	**0.005**
DUXAP9 expression (High vs Low)	1.784 (1.189–2.676)	**0.005**	1.767 (1.178–2.652)	**0.006**

ccRCC, clear cell renal cell carcinoma; TCGA, The Cancer Genome Atlas; HR, hazard ratio; CI, confidence interval. The bold numbers indicate the P-values with statistical significance.

### DUXAP9 Promoted RCC Cell Proliferation and Inhibited Apoptosis

**Figure 3:**
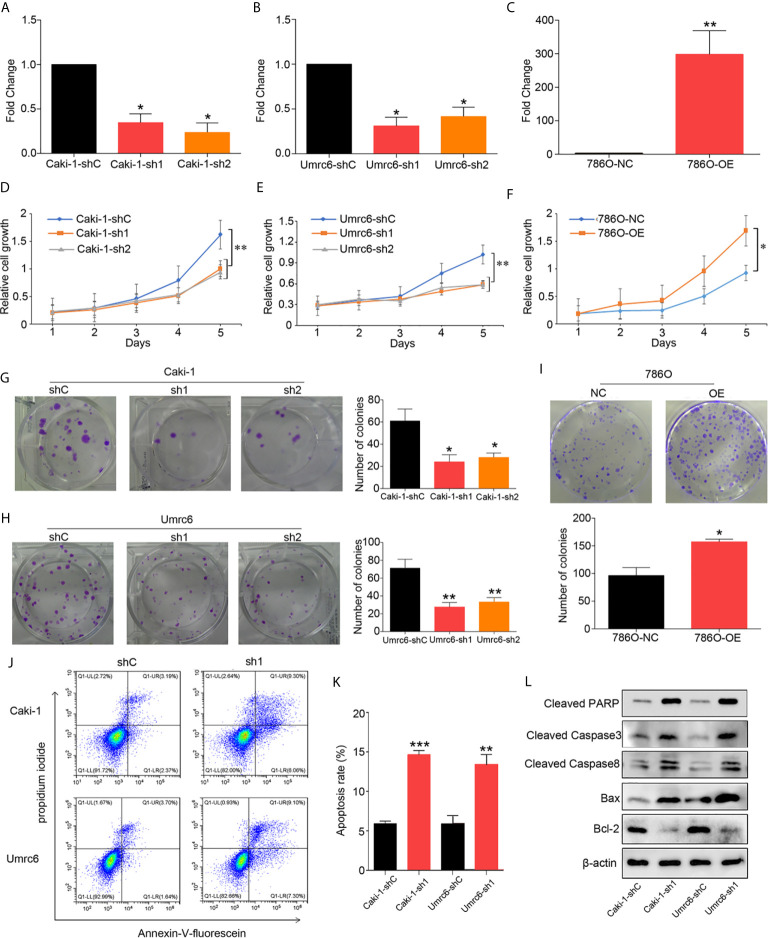
DUXAP9 facilitated proliferation and inhibited apoptosis in renal cancer cells. **(A, B)** DUXAP9 was knocked down in renal cancer cells using shRNA1 or shRNA2 targeting DUXAP9. **(C)** DUXAP9 was overexpressed in the 786O cells (786-O). Proliferation of renal cancer cells after DUXAP9 knockdown or overexpression, as detected by **(D–F)** CCK-8 assays and **(G–I)** colony formation assays. **(J, K)** Apoptosis rate of DUXAP9-knockdown renal cancer cells, as detected by flow cytometry. **(L)** Apoptosis-related proteins in DUXAP9-knockdown renal cancer cells, as detected by western blotting. β-actin was used as the loading control. Data represent mean ± SD from three independent experiments. shRNA, short hairpin RNA; sh1, shRNA1; sh2, shRNA2; shC, shRNA negative control; OE, DUXAP9 overexpression; NC, overexpression negative control; CCK-8, cell counting kit-8; *P < 0.05; **P < 0.01; ***P < 0.001.

To identify the impact of upregulated DUXAP9 in localized ccRCC, we knocked down DUXAP9 using shRNAs targeting DUXAP9 in Umrc6 (17) and Caki-1 cells (**Figures 3A, B** **)** and overexpressed DUXAP9 in 786-O cells (**Figure 3C**). CCK-8 and colony formation assays indicated that DUXAP9 knockdown lead to significant inhibition of Umrc6 and Caki-1 cell proliferation, and DUXAP9 overexpression result in a significant increase in 786-O cell proliferation (P<0.05) (**Figures 3D–I**).

Furthermore, apoptosis changes were observed in DUXAP9 knockdown Umrc6 and Caki-1 cells simultaneously. Therefore, we wondered whether DUXAP9 can modulate apoptosis, resulting in proliferation. Flow cytometry indicated a clearly increased apoptosis rate among the renal cancer cells after DUXAP9 knockdown (**Figures 3J, K** **)**. Furthermore, after DUXAP9 knockdown, Bcl-2 (an anti-apoptosis protein) was markedly decreased, while Bax (a pro-apoptotic protein), cleaved caspase-8, cleaved caspase-3, and cleaved PARP were significantly increased, as detected by western blotting (**Figure 3L**). These results confirmed that DUXAP9 is an important mediator of apoptosis.

### DUXAP9 Promoted RCC Cell Migration and Invasion and Was Involved With EMT

**Figure 4:**
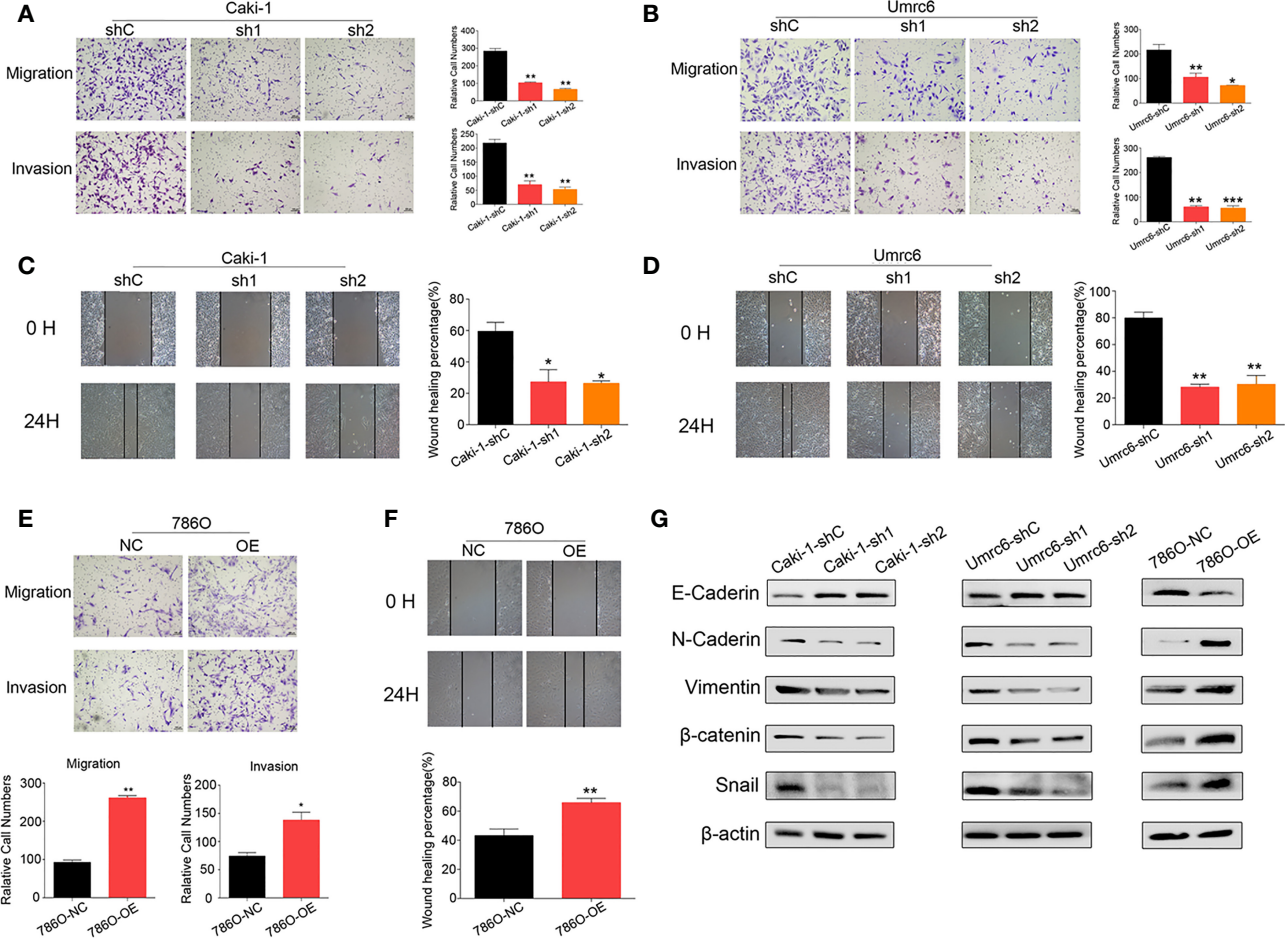
DUXAP9 promoted the aggressive capacity of renal cancer cells and was involved with EMT. **(A, B, E)** Migration and invasion ability, as detected by Transwell assays without/with Matrigel, **(C, D, F)** wound healing assays, and **(G)** expression of EMT-related markers, as detected by western blotting, in renal cancer cells after DUXAP9 knockdown or overexpression. Data represent mean ± SD from three independent experiments. shRNA, short hairpin RNA; sh1, shRNA1; sh2, shRNA2; shC, shRNA negative control; OE, DUXAP9 overexpression; NC, overexpression negative control; EMT, epithelial–mesenchymal transition; *P < 0.05; **P < 0.01; ***P < 0.001.

We further explored the effect of DUXAP9 on RCC in migration and invasion, as our results showed that high DUXAP9 expression was significantly associated with poor PFS in patients with renal cancer cells. DUXAP9 downregulation significantly suppressed the migratory and invasive abilities of Caki-1 and Umrc6 cells *in vitro*, as detected by transwell cell migration/invasion and wound healing assays (**Figures 4A–D**). DUXAP9 overexpression in 786-O cells significantly increased migratory and invasive capacity, compared the control cells (**Figures 4E, F**). As EMT plays a vital role in motility capacity in advanced tumors, we assessed whether DUXAP9 in RCC activates the EMT pathway. As expected, the levels of N-cadherin, vimentin, β-catenin and Snail decreased while the levels of E-cadherin elevated after DUXAP9 knockdown in Caki-1 and Umrc6 cells. On the contrary, after overexpression of DUXAP9 in 786-O cells, the levels of E-cadherin downregulated, whereas the levels of N-cadherin, vimentin, β-catenin and Snail upregulated (**Figure 4G**).

### IGF2BP2 Promotes the DUXAP9 Stability *via* an m6A-Dependent Manner

**Figure 5:**
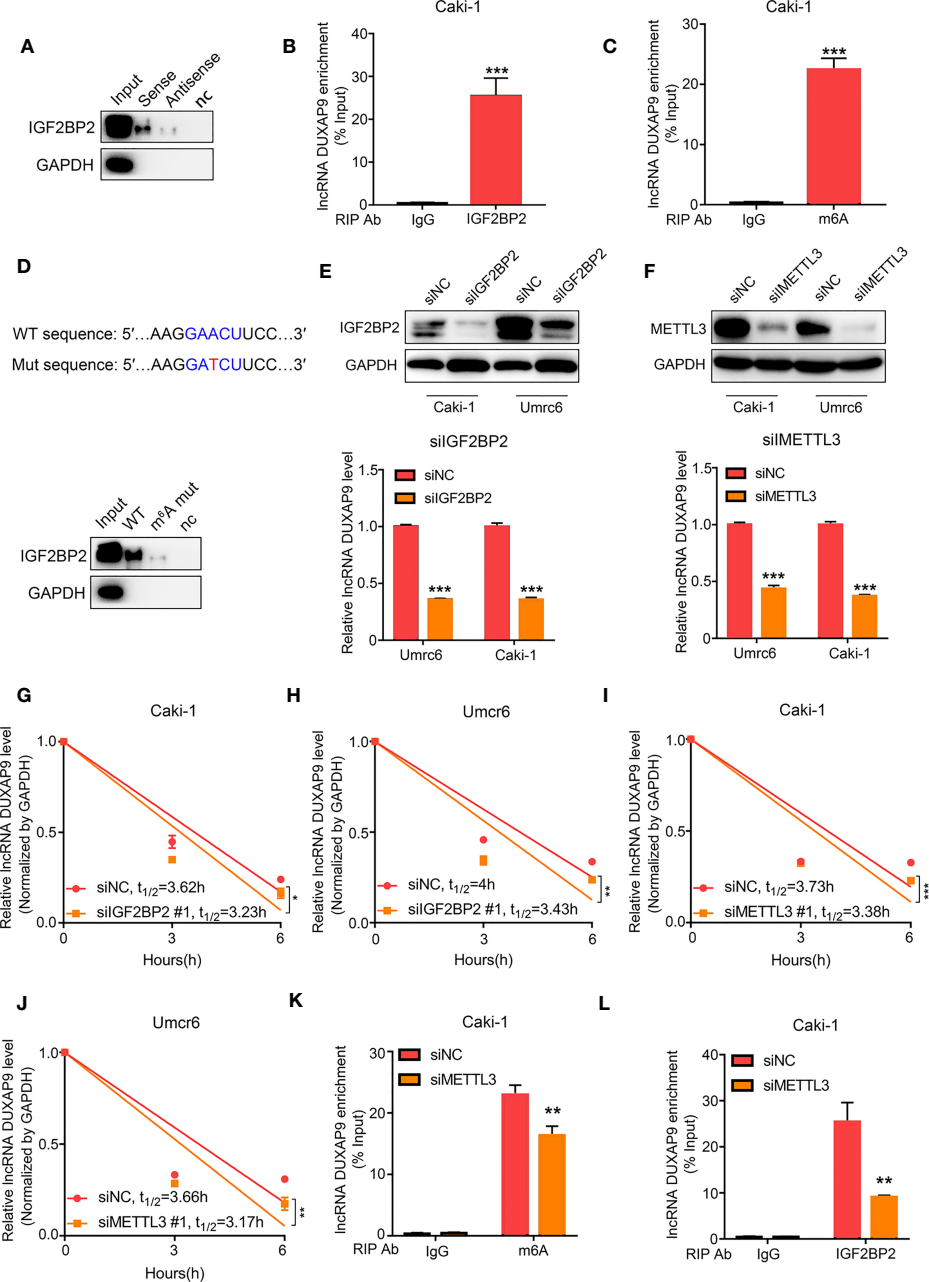
DUXAP9 interacts with IGF2BP2 through GAACU motif (an m6A motif). **(A)** Immunoblotting of IGF2BP2 after RNA pull down assay showing its specific related with DUXAP9 in Caki-1 cells. **(B)** RIP-qPCR assays demonstrated IGF2BP2 binds to DUXAP9. Relative enrichment representing LncRNA levels associated with IGF2BP2 compared to IgG antibody served as a control. **(C)** MeRIP assays in Caki-1 cells confirming that DUXAP9 exhibits m6A modification. **(D)** Top, Schematic diagram of the m6A motif (GAACU) in DUXAP9 and the mutation used in the subsequent RNA pull-down assay. Down: Immunoblotting of IGF2BP2 after RNA pull down assay showed that the binding of IGF2BP2 to DUXAP9 was decreased by the DUXAP9 mutation of the m6A-binding motif. **(E–J)** Inhibition of IGF2BP2 and METTL3 decreased the DUXAP9 level **(E, F)** and the DUXAP9 half-life in Umrc6 **(H, J)** and Caki-1 **(G, I)** treated with actinomycin D to inhibit further transcription. **(K)** MeRIP-qPCR assays (using m6A-specific antibody vs. control) after METTL3 silencing showed that DUXAP9 enrichment was decreased. **(L)** RIP-qPCR assays (using IGF2BP2-specific antibody vs. control) after METTL3 silencing showed that DUXAP9 enrichment was decreased. Data represent mean ± SD from three independent experiments. RIP, RNA immunoprecipitation; qPCR, quantitative polymerase chain reaction; MeRIP, methylated RNA immunoprecipitation; *P < 0.05; **P < 0.01; ***P < 0.001.

To investigate the potential molecular mechanisms of DUXAP9 in regulating RCC, we performed RNA pull-down assays followed by mass spectrometry to identify the proteins that bind to DUXAP9. IGF2BP2 directly bound to DUXAP9 (but not the antisense control) in Caki-1 cells (**Figure 5A**). Furthermore, RIP-qPCR assays validated the interaction between IGF2BP2 and DUXAP9 by confirming the enrichment of DUXAP9 when using IGF2BP2-specific antibody compared to control IgG antibody in Caki-1 cells (**Figure 5B**). IGF2BP2, an RNA-binding protein, is known as an m6A reader that enhances RNA stability in an m6A-dependent manner (18, 19).

A classical m6A motif (GAACT) was found in the DUXAP9 sequence using SRAMP (an m6A modification site predictor), so we wondered whether DUXAP9 exhibited m6A modification, which could maintain its fate in renal cancer cells. Methylated RNA immunoprecipitation (MeRIP) assays and qPCR analysis showed that DUXAP9 was highly enriched compared with the control IgG antibody in Caki-1 cells, confirming the m6A modification in DUXAP9 (**Figure 5C**). Further, mutating the m6A motif in DUXAP9 decreased the binding of IGF2BP2, as detected by pull-down assays (**Figure 5D**). This indicated that the binding of IGF2BP2 to DUXAP9 depends on m6A modification.

To explore whether IGF2BP2 affected the stability of DUXAP9 in RCC, we knocked down IGF2BP2 or METTL3 in Caki-1 and Umrc6 cells and found that the level of DUXAP9 was initially decreased (**Figures 5E, F** **)**. We then treated the IGF2BP2-knockdown cells with the transcription inhibitor actinomycin D and confirmed that the DUXAP9 half-life significantly decreased (**Figures 5G, H** **)**. Meanwhile, when we knocked down METTL3, an essential m6A methyltransferase known as an m6A writer, the DUXAP9 half-lives were markedly shortened upon METTL3 inhibition in Caki-1 and Umrc6 cells (**Figures 5I, J** **)**. In addition, after METTL3 knockdown, DUXAP9 enrichment in RIP-qPCR assays was significantly impaired when using either IGF2BP2- or m6A-specific antibody compared to control IgG antibody in Caki-1 cells (**Figures 5K, L** **)**. These results indicated that IGF2BP2 binds to DUXAP9 and promotes DUXAP9 stability in an m6A-dependent manner in renal cancer cells.

### DUXAP9 Activated the PI3K/Akt Signaling Pathway and Snail Expression in Renal Cancer Cells

**Figure 6:**
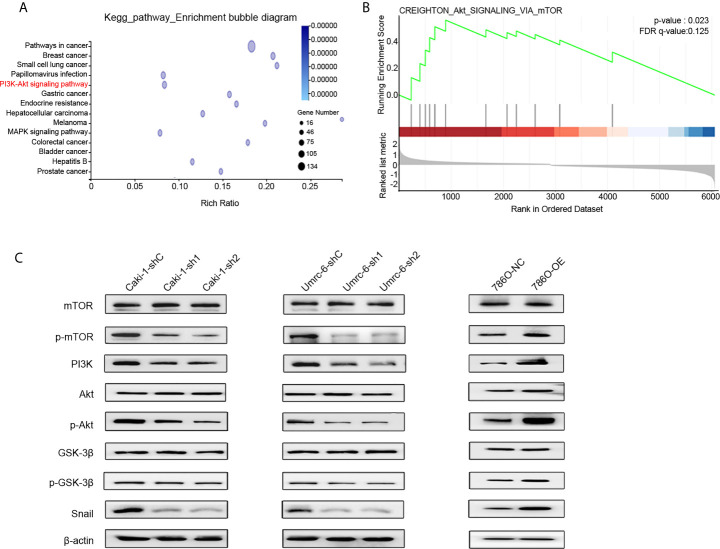
DUXAP9 was involved with the Akt signaling pathway. **(A)** KEGG functional enrichment analysis showing that the differentially expressed genes between DUXAP9 knockdown and control groups were involved in the Akt signaling pathway, as detected by next-generation RNA sequencing of the cells. **(B)** Gene set enrichment analysis identified that DUXAP9 was associated with the Akt signaling pathway, based on RNA sequencing data from 445 localized ccRCC tissues from TCGA database. **(C)** Expression of Akt signaling pathway-related proteins in renal cancer cells in response to DUXAP9 overexpression or knockdown, as detected by western blotting. shRNA, short hairpin RNA; sh1, shRNA1; sh2, shRNA2; shC, shRNA negative control; OE, DUXAP9 overexpression; NC, overexpression negative control; KEGG, Kyoto Encyclopedia of Genes and Genomes; TCGA, The Cancer Genome Atlas.

To identify the potential mechanism of DUXAP9 in RCC, Caki-1 cells with DUXAP9 knocked down and control Caki-1 cells underwent RNA-seq followed by Kyoto Encyclopedia of Genes and Genomes (KEGG) enrichment analysis (**Figure 6A**, **Supplementary Data 2**). We found that DUXAP9 was correlated with the Akt signaling pathway, consistent with the results of the GSEA of TCGA data (**Figure 6B**). It was reported previously that EMT promotion by activation of the Akt signaling pathway plays an essential role in tumor progression (20–22). We explored whether DUXAP9 is involved in Akt-induced EMT, tumor growth, and invasiveness. Western blotting was used to confirm that Caki-1 and Umrc6 cells with DUXAP9 knockdown exhibited downregulated PI3K, p-Akt, and p-mTOR compared to control cells. In contrast, these proteins were upregulated by DUXAP9 overexpression in 786-O cells (**Figure 6C**). Furthermore, p-GSK3β and Snail were downregulated by DUXAP9 knockdown and upregulated by DUXAP9 overexpression, which indicated that DUXAP9 might induce EMT *via* the Akt signaling pathway by Snail.

**Figure 7:**
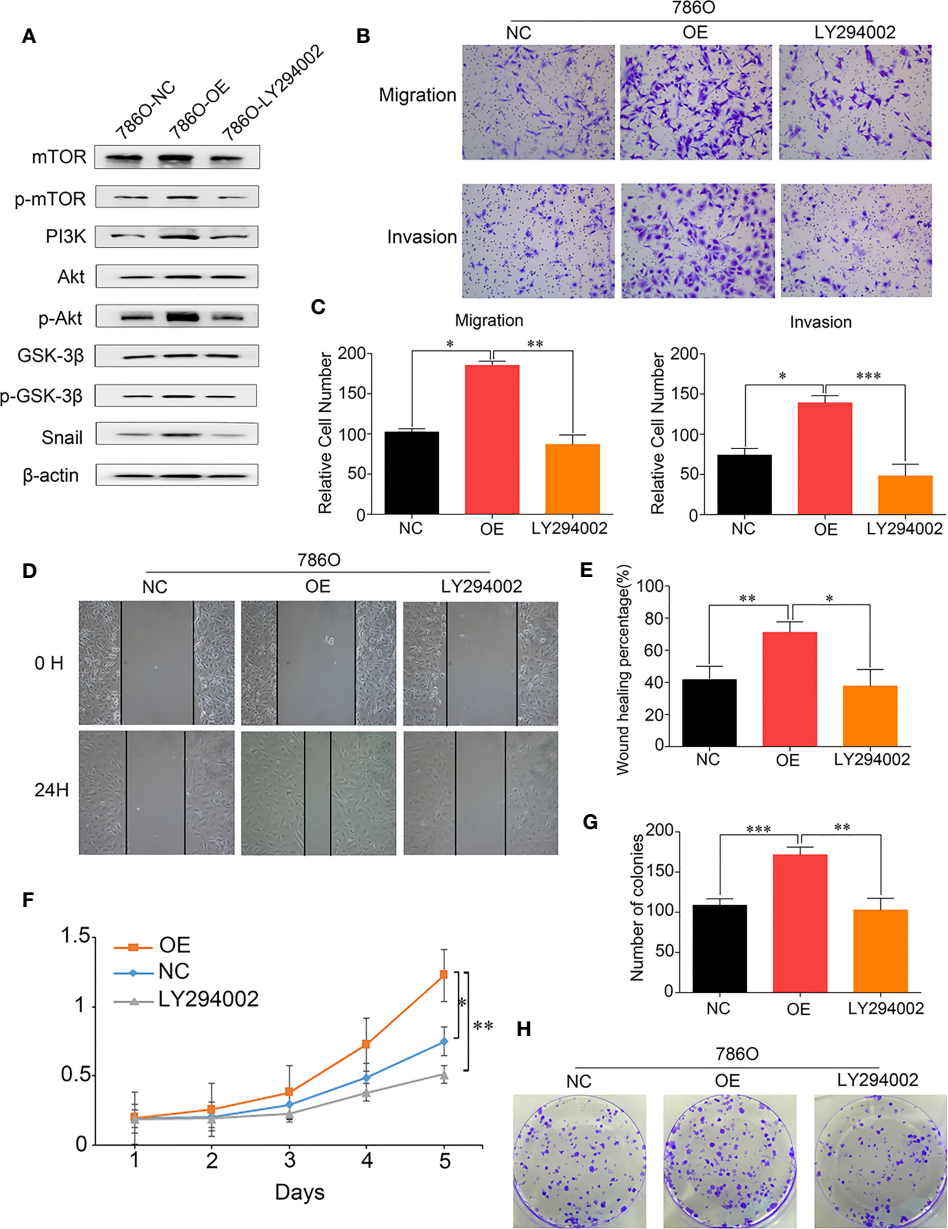
DUXAP9 promoted renal cancer cells invasion, migration and proliferation *via* Akt pathway activation. **(A)** Western blots were performed to evaluate the key Akt pathway proteins levels after overexpression of DUXAP9 treated with or without LY294002. **(B, C)** 786O cells with DUXAP9 overexpression were treated with or without LY294002 and measured through the Transwell chamber without/with Matrigel. **(D, E)** 786O cells with DUXAP9 overexpression were treated with or without LY294002 and subjected to wound‐healing assays. **(F)** 786O cells with DUXAP9 overexpression were treated with or without LY294002 and subjected to CCK-8 assays. **(G, H)** 786O cells with DUXAP9 overexpression were treated with or without LY294002 and subjected to colony formation assays. Data represent mean ± SD from three independent experiments. OE, DUXAP9 overexpression; NC, overexpression negative control; CCK-8, cell counting kit-8; *P < 0.05; **P < 0.01; ***P < 0.001.

To further investigate the mechanism underlying the effects of DUXAP9 on the Akt signaling pathway in RCC, LY294002 (a PI3K inhibitor) was used to try to abrogate the effect of DUXAP9 overexpression on the Akt signaling pathway. The cancer cells were cultured with LY294002 (final concentration 20 μmol/L) for 24 h for further study. Western blotting analysis showed that LY294002 could downregulated the expression levels of p-mTOR, p-Akt, p-GSK3β and Snail previously upregulated accompanied by DUXAP9 overexpression (**Figure 7A**). The transwell cell migration/invasion and wound healing assays validated that implement of LY294002 prominently restricted DUXAP9 overexpression effect on migration and invasion capacities *in vitro* (**Figures 7B–E**). Meanwhile, we next evaluated that management of LY294002 in renal cancer cells also impaired the advancement of tumor cells growth of DUXAP9-overexpression cells through CCK-8 assays and colony formation assays (**Figures 7F–H**).

## Discussion

Previous studies have shown that the recurrence and metastasis of localized ccRCC is a multi-factor event. A variety of genes were altered (1, 3, 23). DUXAP9 has been explored as an oncogene promoting the tumor progression in bladder cancer (10), thyroid cancer (24), non‐small cell lung cancer (25) as well as the growth of renal cell carcinoma (26). In the present study, our results made a more particular knowledge of that lncRNA DUXAP9 is an extremely important factor in promoting the tumor progression of localized ccRCC. Furthermore, we clarified that the stability of DUXAP9 was regulated by m6A modification, and DUXAP9 activated PI3K/Akt signaling pathway and Snail expression in renal cancer cells.

Through RNA pull‐down combined with mass spectrometry, we found that IGF2BP2 bound to the DUXAP9 and this was confirmed using RIP-qPCR assays involving IGF2BP2-specific antibody. A growing body of evidence shows that IGF2BP2 interacts with a variety of lncRNAs to regulate a variety of biological functions (27, 28). Previous studies have shown IGF2BPS belongs to a special m6A card reader family. By identifying and binding to the m6A sequence, they can target thousands of transcripts. m6A modification is associated with many basic biological processes, such as cell proliferation, differentiation, and tumorigenesis (15, 29, 30). We found mutation of the m6A motif (GAACU) in DUXAP9 significantly reduced the IGF2BP2 level in the pull-down assay. Our study is the first to demonstrate that the effects of DUXAP9 rely on m6A modification. It has been well documented that IGF2BP2 promotes the stability and storage of its target RNAs through the m6A-reading process (29, 31). In the present study, this was identified by demonstrating that DUXAP9 levels decreased due to decreased RNA stability after m6A modification was inhibited (by silencing METTL3) as well as after silencing IGF2BP2. These results indicate that DUXAP9 and IGF2BP2 form an RNA–protein complex in renal cancer cells and that the effects of DUXAP9 involve an m6A modification-dependent mechanism.

A previous study by Ting et al. demonstrated that the lncRNA DUXAP9 directly interacts with E3 ubiquitin ligase Cbl-b and reduces the degradation of epidermal growth factor receptor (EGFR). The EGFR signaling pathway was enhanced and induced proliferation and metastasis of non-small cell lung cancer cells (25). To clarify the molecular mechanism underlying the pro-tumor effects of DUXAP9 in renal cancer cells, we did a bioinformatics analysis. Intriguingly, the TCGA data and our RNA-seq data both showed that DUXAP9 was involved in the Akt regulatory network. Of note, Zheng et al. demonstrated that DUXAP9 promoted the proliferation, migration, invasion of thyroid cancer by down-regulating Musashi 2 *via* Akt/Stat3 signaling pathway (24). This is consistent with our findings that DUXAP9 knockdown reduced the activation of Akt signaling in renal cancer cells. Additionally, it has been well documented that the Akt/mTOR pathway is very common in cells, participating in the regulation of diverse processes, such as cell growth and apoptosis (32). Thus, based on the bioinformatics analysis and western blotting, we hypothesized that DUXAP9 exerted proliferation effects by regulating Akt/mTOR. To further validate this hypothesis, we used LY294002 to inhibit PI3K, which abrogated the tumorigenic effects of DUXAP9 in renal cancer cells. The data confirmed that DUXAP9 is an important mediator in the Akt regulatory network, activating PI3K to induce Akt/mTOR signaling in renal cancer cells.

In this study, using western blotting and *in vitro* (cell line) assays, we showed that DUXAP9 is an important regulator of Snail and promotes the invasion and migration in renal cancer cells. These results concur with previous research showing that DUXAP9 promotes metastasis in bladder cancer (10). Further, Snail is a zinc-finger transcriptional repressor that represses epithelial genes and can thereby induce EMT (33). So far, it has been reported that Snail induces EMT in nasopharyngeal carcinoma, bladder cancer, and hepatocellular carcinoma (21, 34). DUXAP9 knockdown decreased Snail expression and inhibited EMT progression, while DUXAP9 overexpression activated Snail-induced EMT signaling in renal cancer cells. Thus, we speculated that DUXAP9 knockdown inhibits EMT at least in part by indirectly decreasing the level of Snail. In cancer, previous studies indicated that Akt activation can trigger EMT by phosphorylating and thereby inhibiting GSK3β, preventing it from phosphorylating Snail and thus preventing Snail nuclear export and degradation (20, 22, 35). In general, we propose hypothetical mechanism that the role of DUXAP9 in promoting EMT involved with the activation of the Akt-GSK3β-Snail signaling pathway.

Nevertheless, there were several limitations to this study. The details mechanisms of DUXAP9 in PI3K/AKT pathway activation remains unclear. And DUXAP9 involved with activity of PI3K/Akt/GSK3β/Snail pathway in ccRCC tumor tissues need further verification. The size of our samples was relatively small, the statistical power may be insufficient. More samples are needed for further verification in the future.

## Conclusion

Herein, we identified the lncRNA DUXAP9 (ENSG00000225210), which upregulated in localized ccRCC. DUXAP9 upregulation was significantly associated with the OS and PFS of localized ccRCC patients as an independent risk factor. DUXAP9 promoted proliferation and motility capacities, suppressed apoptosis, and promoted EMT in renal cancer cells. Further, mechanistic studies showed that m6A modification of DUXAP9 allows it to exert its function by activating the PI3K/Akt signaling pathway and Snail expression in renal cancer cells. Collectively, we revealed the potential prognostic value of DUXAP9 and the potential therapeutic value of developing agents that target DUXAP9 in localized ccRCC.

## Data Availability Statement

The original contributions presented in the study are included in the article/**Supplementary Material**. Further inquiries can be directed to the corresponding authors.

## Ethics Statement

Written informed consent was obtained from the individual(s) for the publication of any potentially identifiable images or data included in this article.

## Author Contributions 

JW, JZC, and JL conceived the study design and performed the experiments. LT, YT, HL, and CG performed the experiments. LT, PL, YY and JJC collected the patient specimens and the data. LT, YT, HL, and JW conducted the data analysis and drafted the manuscript. LT, YT, and HL contributed equally to the work. All authors contributed to the article and approved the submitted version.

## Funding

This study was supported by the National Natural Science Foundation of China (Award Number: 81725016, 81872094, 81772718, 81602219), Guangdong Provincial Science and Technology Foundation of China (Award Number: 2017B020227004, 2017A030313538).

## Conflict of Interest

The authors declare that the research was conducted in the absence of any commercial or financial relationships that could be construed as a potential conflict of interest.
